# Sex-specific Associations Between Type 2 Diabetes Incidence and Exposure to Dioxin and Dioxin-like Pollutants: A Meta-analysis

**DOI:** 10.3389/ftox.2021.685840

**Published:** 2022-02-23

**Authors:** Noa Gang, Kyle Van Allen, Paul J. Villeneuve, Heather MacDonald, Jennifer E. Bruin

**Affiliations:** ^1^ Department of Biology and Institute of Biochemistry, Carleton University, Ottawa, ON, Canada; ^2^ School of Mathematics and Statistics, Carleton University, Ottawa, ON, Canada; ^3^ Department of Public Health Sciences, Queen’s University, Kingston, ON, Canada; ^4^ Health and Biosciences Librarian, MacOdrum Library, Carleton University, Ottawa, ON, Canada

**Keywords:** persistent organic pollutants, type 2 diabetes, dioxins, dioxin-like polychlorinated biphenyls (DL-PCBs), sex differences, meta-analysis

## Abstract

The potential for persistent organic pollutants (POPs), including dioxins and dioxin-like polychlorinated biphenyls (DL-PCBs), to increase the risk of incident diabetes in adults has been extensively studied. However, there is substantial variability in the reported associations both between and within studies. Emerging data from rodent studies suggest that dioxin disrupts glucose homeostasis in a sex-specific manner. Thus, we performed a review and meta-analysis of relevant epidemiological studies to investigate sex differences in associations between dioxin or DL-PCB exposure and type 2 diabetes incidence. Articles that met our selection criteria (n = 81) were organized into the following subcategories: data stratified by sex (n = 13), unstratified data (n = 45), and data from only 1 sex (n = 13 male, n = 10 female). We also considered whether exposure occurred either abruptly at high concentrations through a contamination event (“disaster exposure”) or chronically at low concentrations (“non-disaster exposure”). There were 8 studies that compared associations between dioxin/DL-PCB exposure and diabetes risk in males versus females within the same population. When all sex-stratified or single-sex studies were considered in the meta-analysis (n = 18), the summary relative risk (RR) for incident diabetes among those exposed relative to reference populations was 1.78 (95% CI = 1.37–2.31) and 1.95 (95% CI = 1.56–2.43) for female and males, respectively. However, when we restricted the meta-analysis to disaster-exposed populations, the RR was higher in females than males (2.86 versus 1.59, respectively). In contrast, in non-disaster exposed populations the RR for females was lower than males (1.40 and 2.02, respectively). Our meta-analysis suggests that there are sex differences in the associations between dioxin/DL-PCBs exposure and incident diabetes, and that the mode of exposure modifies these differences.

## Introduction

The incidence of diabetes is increasing worldwide at a rate that cannot be explained solely by genetic predisposition or lifestyle (Knip et al., 2005; Butalia et al., 2016; Franks and McCarthy 2016), prompting investigations into alternative etiological risk factors. There is emerging evidence of a causal association between environmental pollutant exposure and the incidence of type 2 diabetes (T2D) (Carpenter 2008; Hectors et al., 2011; Ngwa et al., 2015). Persistent organic pollutants (POPs) are man-made toxins, released into the environment through industrial, electrical, and agricultural sources (Wikoff, Fitzgerald, and Birnbaum 2012; Hens and Hens 2017). POPs are typically lipophilic, resistant to degradation, and highly mobile, thus leading to ubiquitous global dispersion and bioaccumulation (Fisher 1999). Pollutant exposure can occur abruptly at high levels, as in a disaster event such as an industrial accident or sudden food contamination, but more frequently occurs at chronic low levels (Marinković et al., 2010). Human exposure to POPs occurs typically through the consumption of fish, meat, eggs, and dairy (Schecter et al., 2006; Srogi 2008). Despite global efforts to restrict POP production, use still continues in some countries (Azandjeme et al., 2014; Jaacks et al., 2019) and biomonitoring studies continue to detect POPs in serum and urine of the general population in North America (Haines and Murray 2012; Haines et al., 2017).

### Emerging Links Between Persistent Organic Pollutants and Type 2 Diabetes Pathogenesis

T2D is characterized by insufficient insulin production by pancreatic beta cells in the face of peripheral insulin resistance, which results in chronic hyperglycemia (Porte et al., 2001; Kahn 2003; Weir and Bonner-Weir 2004; Kahn et al., 2009; Chen et al., 2017). This disease manifests slowly and early symptoms of metabolic dysfunction, such as impaired glucose tolerance or hyperinsulinemia, can last for years (Porte et al., 2001; Weir and Bonner-Weir 2004; Chen et al., 2017). Clinical diagnoses of diabetes is internationally defined as glycated haemoglobin (HbA1c) ≥6.5%, fasting plasma glucose ≥7.0 mmol/L, or 2-h plasma glucose during an oral glucose tolerance test (OGTT) of ≥11.1 mmol/L (Gillett 2009). Environmental factors that adversely impact beta cell health and/or peripheral insulin sensitivity could augment underlying vulnerabilities and promote the development of T2D (Franks and McCarthy 2016).

Within the last decade, numerous publications have reported positive associations between exposure to POPs and T2D incidence (Al-Othman et al., 2014; Al-Othman, Abd-Alrahman, and Al-Daghri 2015; Aminov et al., 2016a; Aminov et al., 2016b; Arrebola et al., 2013; Cappelletti et al., 2016; Eslami et al., 2016; Everett and Matheson 2010; Everett and Thompson 2012; Gasull et al., 2012; Han et al., 2020; Huang et al., 2015; Lee et al., 2010; 2011; Lind et al., 2014; Mannetje et al., 2018; Marushka et al., 2017; Patel, Bhattacharya, and Butte 2010; Rahman et al., 2019; Rylander et al., 2015; Singh and Chan 2017; Son et al., 2010; Starling et al., 2014; Suarez-Lopez et al., 2015; Tanaka et al., 2011; Tornevi et al., 2019; Ukropec et al., 2010; Wolf et al., 2019; Wu et al., 2013; Zong et al., 2016). However, the strength of associations between POPs and diabetes incidence varies considerably (Lee et al., 2014; Hectors et al., 2011; Ngwa et al., 2015; Yang et al., 2017). There are many possible explanations for these variations including differences in the type of POPs studied, exposure duration, level of exposure, method of contaminant analysis, underlying health and genetics of the study populations, ascertainment of diabetes diagnosis, and the range of covariates considered. In this meta-analysis, we narrowed down the scope of literature on environmental contaminants by focusing on studies that measured dioxins and dioxin-like (DL) POPs.

### Dioxins and Dioxin-like Pollutants

**FIGURE 1:**
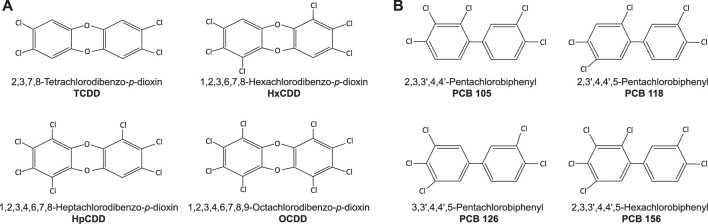
Chemical structure of **(A)** dioxins and **(B)** dioxin-like (DL) PCBs commonly considered in epidemiological studies.

Various chemicals are classified as POPs, including polychlorinated dibenzo-*p*-dioxins and dibenzofurans (PCDD/Fs), polychlorinated biphenyls (PCBs), organochlorine pesticides (OCPs), poly fluorinated alkyl substances (PFAS), and polybrominated diphenyl ethers (PBDEs). Dioxins and DL-PCBs are structurally similar polycyclic, halogenated aromatic chemicals (Figure 1) that share a common mechanism of action via binding to the intracellular aryl hydrocarbon receptor (AhR).

The dioxin family consists of oxygen-linked chlorinated benzene rings that vary in position and number of halogens (Figure 1A). The toxicity of different dioxin compounds is related to their pattern of halogenation. For example, TCDD (2,3,7,8-tetrachlorodibenzo-*p-*dioxin; Figure 1A) is the most toxic and stable dioxin, with a half-life of 7–11 years in humans (Lee et al., 2014; Schecter et al., 2006; Srogi 2008). During much of the 1900s TCDD was produced as a contaminant from industrial processing, but was eventually recognized as a health hazard to industrial workers (Vena et al., 1998; Calvert et al., 1999; Steenland et al., 1999; Cappelletti et al., 2016) and local communities (Cranmer et al., 2000; Karouna-Renier et al., 2007; MacNeil et al., 2009; Chang et al., 2010; Ukropec et al., 2010; Karnes, Winquist, and Steenland 2014; Huang et al., 2015). Widespread distribution of TCDD occurred during the Vietnam war when Agent Orange, a defoliate contaminated with TCDD, was used by the United States military as part of a chemical warfare program known as Operation Ranch Hand (Henriksen et al., 1997; Kim et al., 2003; Longnecker and Michalek 2000; Michalek and Marian, 2008; Steenland et al., 2001). Furthermore, in 1976 an industrial disaster in Seveso, Italy released tonnes of nearly pure TCDD gas into the local regions. Other dioxins commonly considered within the literature and included in our review are 1,2,3,6,7,8-Hexachlorodibenzo-*p*-dioxin (HxCDD), 1,2,3,4,6,7,8-Heptachlorodibenzo-*p*-dioxin (HpCDD), and 1,2,3,4,6,7,8,9-Octachlorodibenzo-*p*-dioxin (OCDD) (Figure 1A). However, TCDD has been more widely studied for sub-lethal exposure effects compared to other dioxins, as it was involved in more disaster and occupational exposure events (Birnbaum and Couture 1988; Couture, Elwell, and Birnbaum 1988; Rozman 1999; Schwetz et al., 1973; Viluksela et al., 1997; 1998).

The PCB family includes 209 separate congeners, 12 of which are considered DL-PCBs (US Environmental Protection Agency 2003) (Figure 1B). PCBs are exceptionally stable, heat resistant and non-flammable, and were intentionally manufactured between the 1920s to 70s for electrical components, hydraulic fluids, lubricants, and industrial insulating or heat-exchange fluids (White and Birnbaum 2009; Wikoff, Fitzgerald, and Birnbaum 2012). Disaster exposure to DL-PCBs occurred in Michigan, United States, in 1973 when animal feed accidently contaminated with PCBs was distributed to farms (Vasiliu, 2006). Additionally, between 1978 and 1979, rice-bran oil contaminated with PCBs and PCDFs poisoned thousands of inhabitants of Yucheng, Taiwan with “oil disease” (Wang et al., 2008). These incidents of disaster exposure to DL-PCBs, albeit far from isolated events, are the most widely studied when considering pathologies related to metabolic diseases (Taylor, 2001; Hens and Hens 2017).

### Evidence for a Causal Link Between Dioxin Exposure and Diabetes

Dioxins and DL-PCBs act as ligands for AhR, leading to the upregulation of target genes such as cytochrome P450 (*Cyp*)*1a1*. Our lab showed that TCDD-exposed mice have persistent CYP1A1 upregulation in pancreatic islets, where insulin-secreting beta cells reside, a sign that TCDD reaches the endocrine pancreas *in vivo* (Ibrahim et al., 2020). A single high-dose injection of TCDD in mice caused hypoinsulinemia *in vivo* for up to 6 weeks and reduced glucose-stimulated insulin secretion in islets *ex vivo* (Hoyeck et al., 2020; Ibrahim et al., 2020)*.* Furthermore, direct TCDD exposure *in vitro* caused *CYP1A1* upregulation and suppressed glucose-induced insulin secretion in both mouse and human islets (Kurita et al., 2009; Ibrahim et al., 2020). These data suggest that TCDD may be driving metabolic dysfunction, at least in part, via direct effects on pancreatic islets.

There is also emerging evidence that female rodents are more susceptible to the diabetogenic effects of dioxins compared to their male counterparts (Naville et al., 2013; Hoyeck et al., 2020; Matteo et al., 2021). For example, while a single high-dose injection of TCDD at 8 weeks of age caused persistent hypoinsulinemia in both male and female mice *in vivo*, only male mice had increased insulin sensitivity and altered islet cell composition, and only female mice developed transient hyperglycemia following TCDD exposure (Hoyeck et al., 2020). Consistent with the single high-dose model, repeated low-dose TCDD exposure for 12 weeks starting at 6–8 weeks of age both exacerbated and accelerated the onset of high fat diet (HFD)-induced glucose intolerance in female but not male mice (Matteo et al., 2021). Naville et al. (2013) also observed exacerbated HFD-induced glucose intolerance in female, but not male mice, exposed chronically from 5 to 10 weeks of age to a low-dose mixture of pollutants that included TCDD.

Considering the sex differences reported in mouse models, we hypothesized that there are sex differences in the strength of the association between dioxin/DL pollutant exposure and T2D incidence in humans. To explore this question, we performed a meta-analysis of epidemiological studies that assessed exposure to either dioxins or DL-PCBs and the incidence of T2D or metabolic syndrome. We also considered the mode of pollutant exposure, where a disaster event (e.g., Seveso) reflects an abrupt, high-dose exposure versus a non-disaster chronic exposure scenario that includes both low level exposure in the general population and high-level exposure in occupational settings. Importantly, abrupt exposure via disaster can lead to a prolonged period of high pollutant concentration in the body due to the long half-life of dioxins and DL pollutants (Fisher, 1999).

## Methods

### Literature Search Strategy

We conducted a literature search for studies examining POPs, dioxins, or benzofurans and diabetes in humans via PubMed on May 17, 2021. MeSH terms, substance registry numbers, and keywords were used. The search terms can be found in Supplementary Table S1. Review articles were excluded unless original data were presented. A filter for human studies was applied for studies published before 2020. A simple secondary search was conducted in Google Scholar to supplement the PubMed search. Our literature search strategy does not meet the criteria for a systematic literature review as only one database (PubMed) was used to conduct our primary literature search, with some supplementation from Google Scholar. However, our literature search and formal inclusion/exclusion criteria does provide sufficient data for the purposes of a comprehensive meta-analysis.

Articles were screened by two independent reviewers (NG and KVA) using the following inclusion criteria: the population included human adults exposed to dioxins, DL-PCBs, or benzofurans, and an outcome of T2D, hyperglycemia, prediabetes, metabolic syndrome, or glucose intolerance.

### Data Extraction and Study Quality Assessment

Data extracted from each study included: authors, publication year, cohort location, type of study design (e.g., cohort, cross-sectional, case-control), sample size, type of exposure (disaster or non-disaster), specific pollutant(s) measured, diabetes assessment method, outcome determined, considerations made for sex-specific associations (whether data was stratified by sex, unstratified by sex, or only considered 1 sex), analysis strategy (e.g., control for confounding variables), and measures of association (see Table 1 for key details and Supplementary Table S2 for additional information on each study).

**TABLE 1:** Summary of the 18 articles included in the meta-analysis. These studies investigated the association between exposure to dioxins/DL-PCBs and incidence of type 2 diabetes (T2D) or metabolic syndrome. The measure of association represents exposed populations relative to reference populations with undetectable levels of contaminants. CI = confidence interval, DL = dioxin-like, PCB = polychlorinated biphenyl, RR = relative risk, OR = odds ratio, IRR = incidence rate ratio, IDR = incidence density ratio.

	References	Study Population	Outcome	Pollutant(s)	Mode of exposure	Measure of Association (95% CI)
Sex-stratified studies (8 articles)	Bertazzi et al. (2001)	Males (n = 22,607)	T2D	TCDD	Disaster (plant explosion)	RR = 0.6 (0.1–4.1) males
		Females (n = 22,762)				RR = 2.2 (1.0–4.6) females
	Han et al. (2020)	Males (n = 151)	T2D	PCB-118	Non-disaster (low level)	OR = 4.62 (1.52–14.06) males
		Females (n = 165)				OR = 3.08 (0.92–10.35) females
	Huang et al. (2015)	n = 2,898 (total participants)	T2D	PCDD/Fs	Non-disaster (high level)	OR = 3.3 (2.0–5.5) males
						OR = 2.3 (1.2–4.4) females
	Huang et al. (2017)	Males (n = 1,453)	Metabolic Syndrome	PCDD/Fs	Non-disaster (high level)	OR = 1.59 (1.22–2.08) males
		Females (n = 1,305)				OR = 1.16 (0.8–1.68) females
	Silverstone et al. (2012)	Males (n = 153)	T2D	DL-PCBs	Non-disaster (high level)	OR = 0.71 (0.36–1.39) males
		Females (n = 427)				OR = 1.51 (1.02–2.24) females
	Turyk et al. (2009)	Males (n = 279)	T2D	PCB-118	Non-disaster (low level)	IRR = 1.40 (0.50–4.20) males
		Females (n = 192)				IRR = 1.10 (0.20–5.30) females
	Vasiliu, (2006)	Males (n = 688)	T2D	Total PCBs	Disaster (contaminated animal feed)	IDR = 1.74 (0.91–3.34) males
		Females (n = 696)				IDR = 2.33 (1.25–4.34) females
	Wang et al. (2008)	Males (n = 307)	T2D	Mixed DL-PCBs	Disaster (contaminated rice-oil bran)	OR = 1.7 (0.7–4.3) males
		Females (n = 441)				OR = 4.6 (1.9–11.4) females
Male-only studies (7 articles)	Cappelletti et al. (2016)	n = 363	T2D	PCDD/Fs	Non-disaster (high level)	RR = 2.39 (1.67–3.41)
	Kang et al. (2006)	n = 2,927	T2D	TCDD	Non-disaster (high level)	OR = 1.49 (1.10–2.02)
	Kim et al. (2003)	n = 1,378	T2D	TCDD	Non-disaster (high level)	OR = 2.69 (1.09–6.67)
	Michalek and Pavuk (2008)	n = 2,469	T2D	TCDD	Non-disaster (high level)	RR = 1.58 (1.12–2.24)
	Persky et al. (2012)	n = 63	T2D	DL-PCBs	Non-disaster (high level)	OR = 2.7 (1.3–5.8)
	Steenland et al. (2001)	n = 2,759	T2D	TCDD	Non-disaster (high level)	OR = 3.21 (1.81–5.72)
	Yamamoto et al. (2015)	n = 678	T2D	PCDD/Fs	Non-disaster (high level)	OR = 4.98 (1.17–21.17)
Female-only studies (3 articles)	Berg et al. (2021)	n = 88	T2D	DL-PCBs	Non-disaster (low level)	OR = 2.24 (1.0–5.0)
	Rylander et al. (2015)	n = 212	T2D	PCB-118	Non-disaster (low level)	OR = 1.55 (0.41–5.86)
	Zong et al. (2018)	n = 1,586	T2D	DL-PCBs	Non-disaster (low level)	OR = 1.36 (1.01–1.84)

Study quality was assessed by two independent assessors (NG and KVA) using the Newcastle-Ottawa Quality Assessment Scale (Supplementary Table S2). We performed a meta-regression to evaluate whether summary relative risks varied by study quality (moderate vs high quality) across studies (Supplementary Figures S1,S2). Finally, the published measures of association included in the meta-analysis were corrected for publication bias via funnel plot with Trim and Fill, as well as Egger’s test (Egger et al., 1997; Shi and Lin, 2019) (Supplementary Figures S3–S6).

Primary summary measures were usually stated as relative risk (RR) or odds ratio (OR), however incidence rate ratio (IRR), and incidence density ratio (IDR) association measures were also included. We considered these measures of association to be interchangeable and therefore, changed OR, IDR, IRR values to RR as an approximation of the risk estimates.

### Meta-Analysis Strategy

All forest plots included in the meta-analysis were accompanied with *I* ^*2*^-statistics to quantify heterogeneity in the measures of association across studies (Borenstein et al., 2009). All analyses were performed using a random-effects model (DerSimonian and Laird) with OR/RR/IDR/IRR summary measures and 95% confidence intervals (CIs).

We first performed grouped analysis combining both sexes in all stratified and non-stratified studies (n = 18). Then, sex-specific associations between contaminant exposure and T2D incidence were evaluated using sub-group analysis via forest plot for male and female populations separately (n = 15 and n = 11, respectively). Lastly, to assess sex-specific susceptibility to T2D within a study population, we modeled the difference in the measures of association [Female (RR)] — [Male (RR)] in each sex-stratified study to generate a summary risk difference (RD) along with the corresponding standard error for this difference. For males, the logarithm of the relative risk was first calculated from the published value as follows: *β_m_* =*ln*(*RR*). The standard error of this estimate was obtained from the published 95% CI. This was done by first converting the lower and upper published 95% CIs onto the natural logarithm scale and calculating the difference between these transformed values. This distance represents 3.92×*s.e.*(*β_m_*). We then repeated this for the female relative risks. The risk difference, on the log scale, was thus *β*_*f*_–*β_m_* and the standard error of this difference was derived with the formula:

$$s.e.\sqrt{\beta_{f}-\beta_{m}}=\sqrt{s.e.{\left(\beta m\right)}^{2}+s.e.{\left(\beta f\right)}^{2}}$$

All forest plots and meta-regressions were presented on a log scale.

We also calculated the summary measures of association across mode of exposure (non-disaster versus disaster), type of pollutant, and geographical location (continent). “Non-disaster exposure” was defined as long-term low-level exposure typical of the general population (for example through consumption of high fat animal products), chronic moderate-level residential exposure in a contaminated region, or long-term high-level occupational exposure (typically military or industrial). “Disaster-exposure” was defined as abrupt, high-concentration exposure from sudden release of pollutant(s), as through industrial accident or food contamination.

All analyses were performed using the STATA 17SE statistical software (StataCorp. 2019. *Stata Statistical Software: Release 16*. College Station, TX: StataCorp LLC).

## Results

### Summary of Articles

**FIGURE 2:**
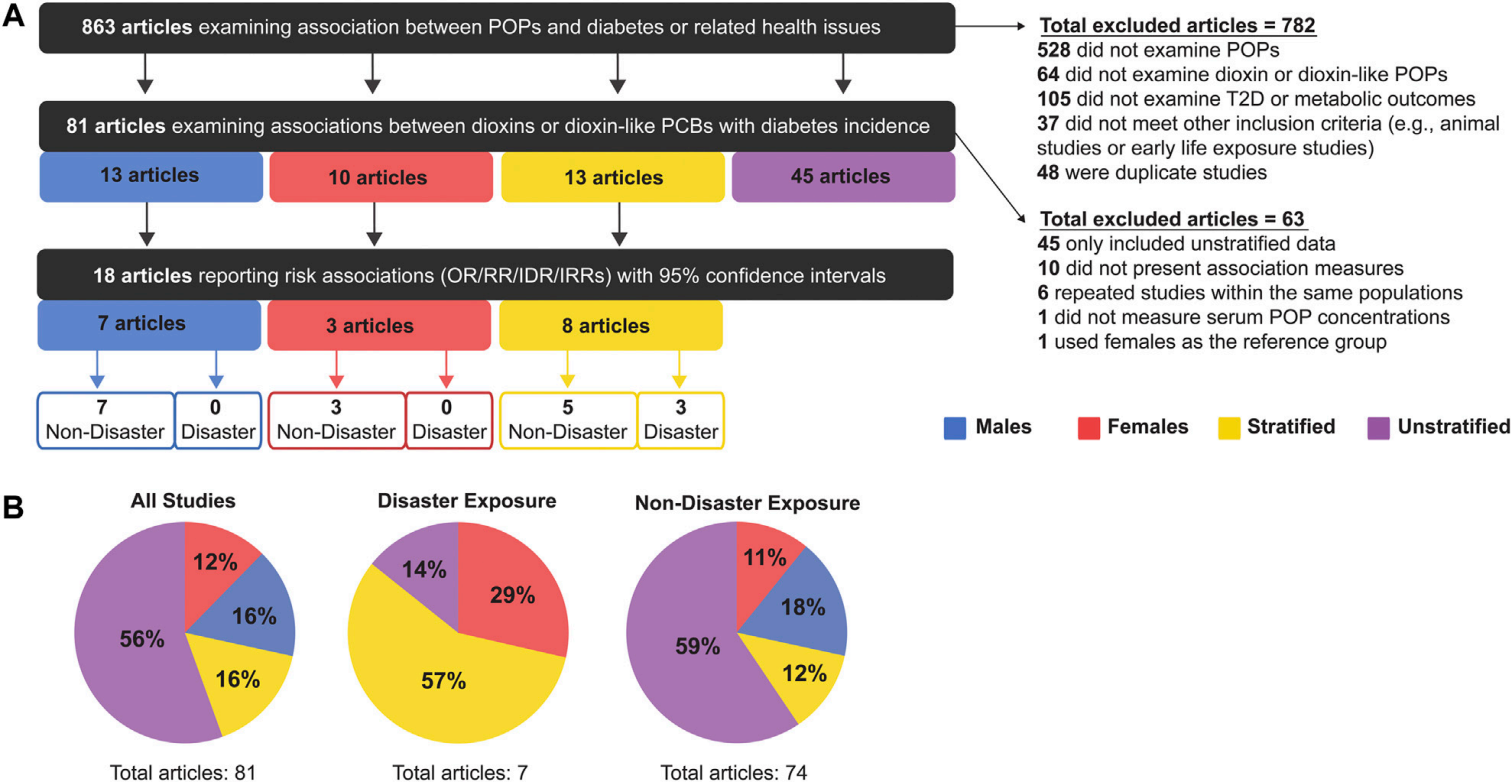
**(A)** Summary of inclusion and exclusion criteria for articles analysed in this meta-analysis, and **(B)** the proportion of studies that were sex-stratified, sex-unstratified, or considered male-only or female-only populations.

Our literature search identified 863 articles, of which 81 met our inclusion criteria (Figure 2). Studies were excluded based on the following criteria: 1) studies that did not examine POPs (528 articles); 2) studies that did not examine dioxins or DL POPs (64 articles); 3) studies that did not examine T2D or related metabolic outcomes (105 articles); 4) animal studies or early life exposure studies (37 articles); 5) duplicate studies (48 articles). The age range of study populations in the 81 included studies was 20–59 years (Supplementary Table S2).

Of the 81 articles that met our initial inclusion criteria, the majority (56%, 45 articles) presented sex-unstratified data (i.e. combined male and female) and only 13 studies stratified data by sex (Figure 2). There were also 13 studies with data from males only and 10 studies with data from females only (Figure 2A). We proceeded to perform our meta-analysis on the 18 articles that either stratified data by sex or reported data from 1 sex and also reported measures of association (Bertazzi et al., 2001; Han et al., 2020; Huang et al., 2015, 2017; Silverstone et al., 2012; Turyk et al., 2009; Vasiliu, 2006; Wang et al., 2008; Cappelletti et al., 2016; Kang et al., 2006; Kim et al., 2003; Michalek and Pavuk, 2008; Persky et al., 2012; Steenland et al., 2001; Yamamoto et al., 2015; Berg et al., 2021; Rylander et al., 2015; Zong et al., 2018). Studies were excluded from the meta-analysis if they 1) presented data that were not stratified by sex (45 articles) 2) did not report measures of association (i.e., OR/RR/IDR/IRR) (10 articles); 3) repeated measures within an already studied population (of these, only the most recent study was included-6 articles); 4) did not determine exposure by serum pollutant concentrations (1 article); and 5) did not use an appropriate reference group (1 article) (Figure 2A). All studies reported clinical or self-reported T2D or metabolic syndrome diagnosis; most studies of the latter category medically verified self-reported cases.

We found evidence of publication bias using funnel plots and Egger’s test (*p* < 0.05) (Supplementary Figure S3, S4). However, this finding was driven by the female data (*p* = 0.025). We corrected for this publication bias using the Trim and Fill method, and while the effect size was reduced slightly, the overall relative risk of diabetes incidence in exposed females remained significantly increased compared to reference populations (Supplementary Figure S5). Male-only data showed no evidence of publication bias (Supplementary Figure S6).

The 81 studies were subsequently categorized by mode of chemical exposure. Of the studies conducted on disaster-exposed populations (n = 7), 57% were stratified by sex, 29% contained female-only data, 14% were unstratified and none examined male-only data (Figure 2B). In contrast, for studies examining non-disaster exposure to dioxin or DL-PCBs (n = 74), the majority reported sex-unstratified data (59%), followed by male-only data as the second most predominant (18%), and sex-stratified or female-only data composing only 12 and 11% of studies, respectively (Figure 2B). Of the final 18 articles included in our meta-analysis, 15 articles were non-disaster exposure (7 male-only, 3 female-only, 5 stratified) and 3 articles were disaster exposure, all of which reported sex-stratified data (Figure 2).

### Both Sexes Show a Significant Association Between Pollutant Exposure and Type 2 Diabetes

**FIGURE 3:**
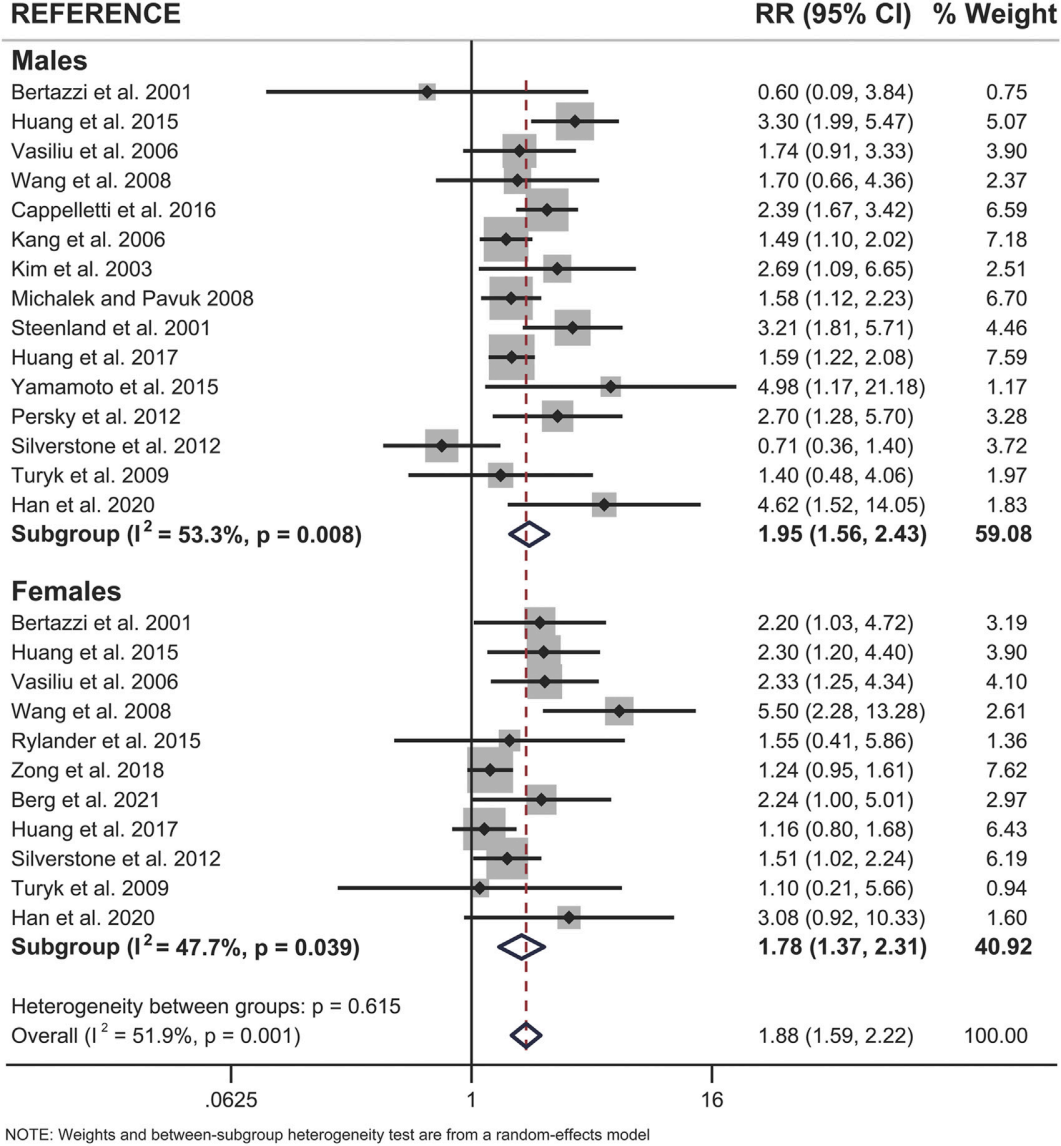
Forest plot of all studies included in the meta-analysis (n = 18), separated by sex. The relative risk (RR) of type 2 diabetes incidence is reported for populations exposed to dioxin/DL-PCBs compared to reference populations with undetectable levels of contaminants. The RR is provided for each study, along with the calculated summary effect measure for each subgroup (males, females). Effect measures are presented on a natural logarithm scale. CI = confidence interval.

**FIGURE 4:**
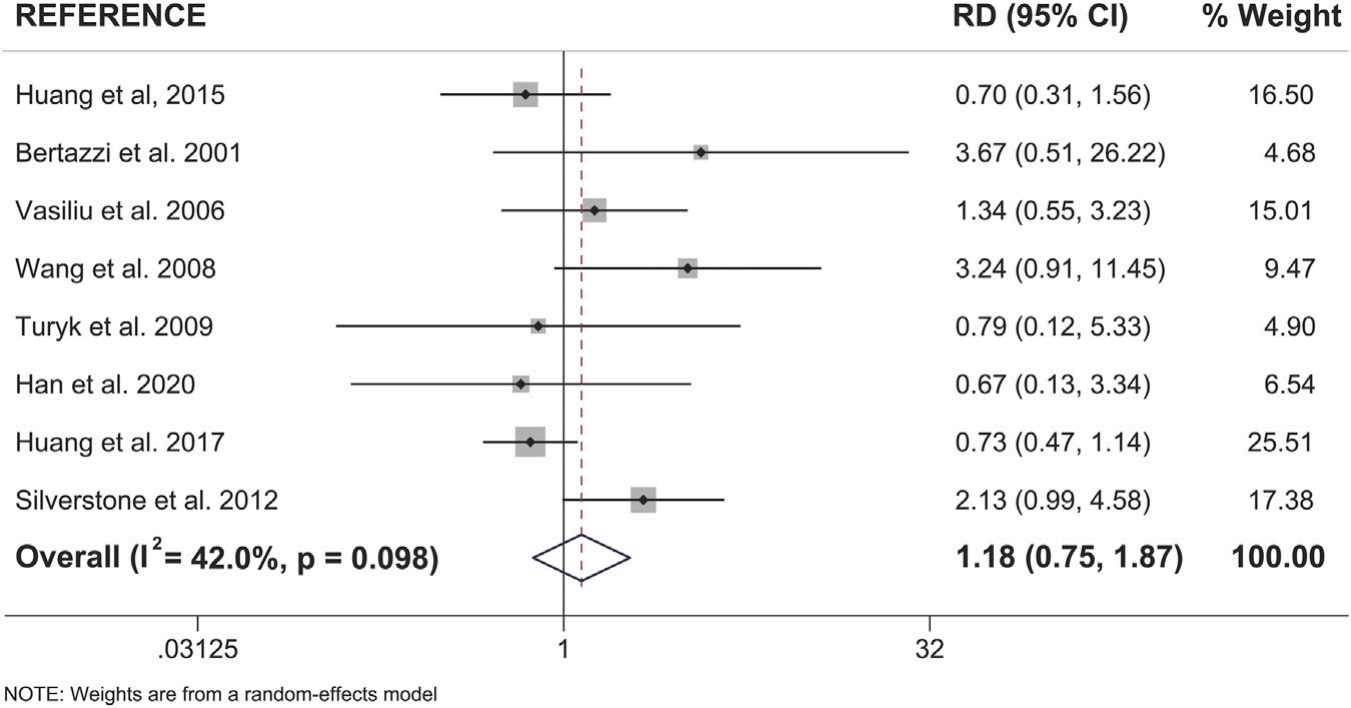
Forest plot of female versus male risk difference (RD) for the association of dioxin/DL chemical exposure and diabetes incidence within the same study population. Summary RD measures were generated from the difference in summary effect measures between the sexes (Females - Males) along with the corresponding standard error for this difference. Effect measures (RDs) are presented on a natural logarithm scale. CI = confidence interval.

When examining pooled data from all 18 studies, we found that both sexes showed a statistically significant positive association between pollutant exposure and T2D (Figure 3; Table 2). Males showed a 1.95x increased summary risk (95% CI = 1.56–2.43) between exposed and reference populations and females showed a 1.78x increased risk (95% CI = 1.37–2.31) between exposed and reference populations (Figure 3). There was also no significant difference between sexes when we examined the calculated RD (females - males) within studies that reported sex-stratified data (RD = 1.18, 95% CI = 0.75–1.87) (Figure 4).

**TABLE 2:** Sex-specific associations between dioxin/DL chemical exposure and diabetes outcome, segregated by type of pollutant, mode of exposure, and continent. The measure of association summary estimate (relative risk, RR) for each subgroup compares exposed populations relative to reference populations with undetectable levels of contaminants. Effect measures (RRs) are presented on a natural logarithm scale. CI = confidence interval.

		*Heterogeneity*			*Summary Estimates*	
		n	I^2^	*p*-value	RR	95% CI
***Males***	**Type of Pollutant**	—	—	—	—	—
	Overall	15	51.3%	0.008	1.95	1.56–2.43
	TCDD	5	48.6%	0.100	1.84	1.31–2.58
	PCDD/Fs	4	66.8%	0.029	2.35	1.57–3.52
	Total PCBs	1	n/a	n/a	1.74	0.91–3.33
	DL-PCBs	3	71.4%	0.030	1.45	0.63–3.38
	PCB-118	2	56.7%	0.128	2.51	0.78–8.10
	**Mode of Exposure**					
	Overall	15	53.3%	0.008	1.95	1.56–2.43
	Disaster	3	0%	0.562	1.59	0.95–2.66
	Non-disaster	12	61.4%	0.003	2.02	1.58–2.59
	**Continent**	—	—	—	—	—
	Overall	15	53.3%	0.008	1.95	1.56–2.43
	Asia	6	53.8%	0.055	2.49	1.62–3.81
	Europe	2	51.3%	0.152	1.64	0.49–5.48
	North America	7	55.1%	0.037	1.67	1.22–2.27
***Females***	**Type of Pollutant**	—	—	—	—	—
	Overall	11	47.7%	0.039	1.78	1.37–2.31
	TCDD	1	n/a	n/a	2.20	1.03–4.72
	PCDD/Fs	2	68.9%	0.073	1.55	0.80–3.00
	Total PCBs	1	n/a	n/a	2.33	1.25–4.34
	DL-PCBs	4	73.4%	0.010	1.89	1.16–3.09
	PCB-118	3	0.0%	0.569	1.91	0.87–4.20
	**Mode of Exposure**	—	—	—	—	—
	Overall	11	47.7%	0.039	1.78	1.37–2.31
	Disaster	3	32.7%	0.226	2.86	1.70–4.84
	Non-disaster	8	3.1%	0.406	1.40	1.17–1.68
	**Continent**	—	—	—	—	—
	Overall	11	47.7%	0.039	1.78	1.37–2.31
	Asia	4	76.3%	0.005	2.38	1.15–4.92
	Europe	3	0.0%	0.887	2.10	1.26–3.51
	North America	4	16.9%	0.307	1.44	1.12–1.84

Note: n = number of studies included for each subgroup analysis. CI = confidence interval.

We next looked for sex differences when studies were further subcategorized by population location or type of pollutant (Table 2). There were no clear sex differences within either of these subcategories, with the exception of DL-PCBs, which showed a significant association with T2D in females (RR = 1.89, 95% CI = 1.16–3.09; 4 studies), and an association that was attenuated in their male counterparts (RR = 1.45, 95% CI = 0.63–3.38; 3 studies) (Table 2). However, most of these subcategories had too few studies to assess sex differences.

### Sex Differences for Risk by Mode of Exposure

**FIGURE 5:**
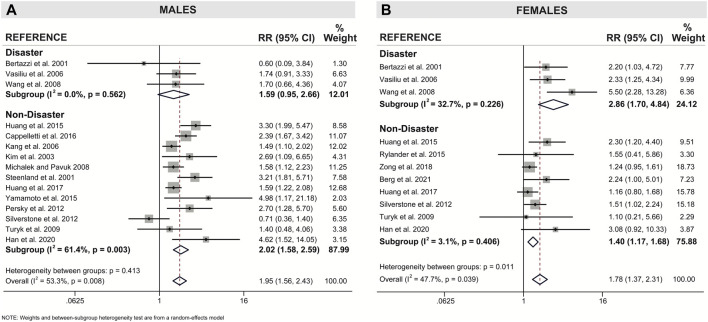
Forest plot of association between dioxin/DL chemical exposure and diabetes incidence with separate subgroup analysis for disaster and non-disaster exposure in **(A)** males and **(B)** females. The relative risk (RR) is provided for each study, along with the calculated summary effect measure. Effect measures (RRs) are presented on a natural logarithm scale. CI = confidence interval.

Our meta-analysis revealed clear sex-specific associations when we categorized studies by mode of exposure (i.e., disaster versus non-disaster). Males exposed to pollutants via non-disaster showed a significant 2.02x increased risk for T2D incidence relative to reference populations (95% CI = 1.58–2.59), compared to a non-significant 1.59x increased risk when exposed via disaster (95% CI = 0.95–2.66) (Figure 5A; Table 2). Females show the opposite trend, with a modest 1.40x increased risk associated with non-disaster exposure (95% CI = 1.17–1.68) compared to a pronounced 2.86x increased risk associated with disaster exposure (95% CI = 1.70–4.84) (Figure 5B; Table 2). Therefore, in disaster exposure conditions, the increased risk for T2D is primarily driven by a significant increase in the RR for females but not males. To assess whether study quality confounded the resulting effect measures we performed meta-regression with study quality as the covariate and found no significant contribution of study quality to the final summary effect measure (Supplementary Figures S1, S2).

**FIGURE 6:**
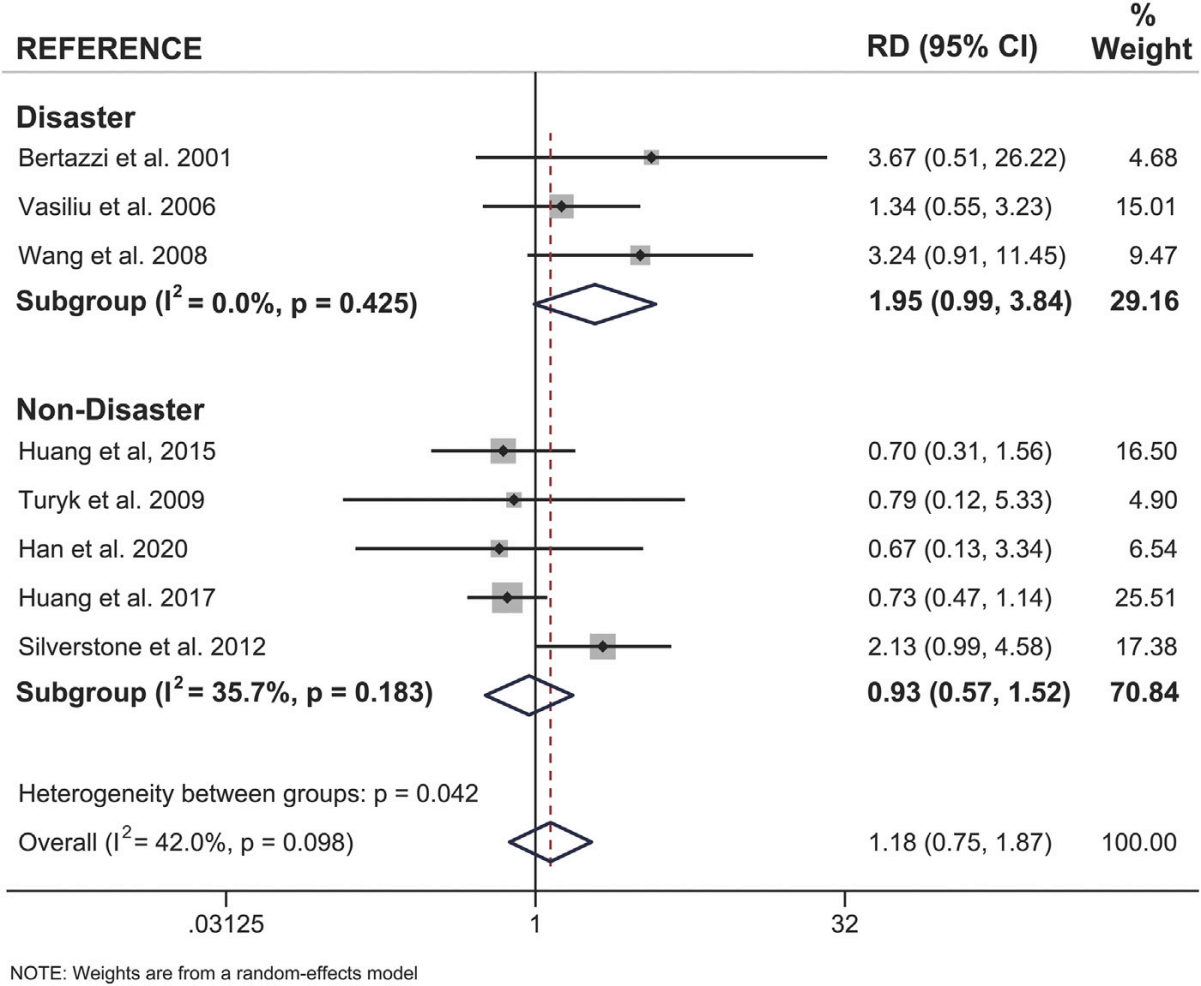
Forest plot of female versus male risk difference (RD) for dioxin/DL chemical exposure, segregated by disaster and non-disaster subgroup analysis, and diabetes incidence within the same study population. Summary RD measures were generated from the difference in summary effect measures between the sexes (Females - Males) and stratified by mode of exposure. Effect measures (RDs) are presented on a natural logarithm scale. CI = confidence interval.

To further investigate sex differences, we performed a meta-analysis on only sex-stratified studies using the calculated differences in association measures between the sexes within the same population (Figure 6). As expected, there was no difference in the risk difference between the sexes when considering non-disaster exposure studies (RD = 0.93, 95% CI = 0.57–1.52). There was an approximately 2-fold increase in risk difference between the sexes when only disaster-exposure studies were analyzed (RD = 1.95; 95% CI = 0.99–3.84) (Figure 6).

## Discussion

A total of 18 articles were included in this meta-analysis that investigated whether there are sex-specific associations between dioxin or DL-PCB exposure and T2D incidence in adults. When we considered all studies together, there were no meaningful sex differences in this association, but differences emerged when data were stratified by mode of exposure. Specifically, we found that the strength of association between exposure to dioxin/DL-PCBs via disaster and incident diabetes was stronger in females than males. Importantly, this sex difference was consistent when we calculated the summary RR for females versus males as well as the risk difference within studies where sex-stratified data was presented. To our knowledge, mode of exposure has not previously been considered when examining sex differences in epidemiology literature on pollutant exposure.

Our review of the epidemiological literature revealed important gaps that limited our meta-analysis of sex-specific associations between pollutant exposure and diabetes. Sex-stratified studies accounted for only 16% of the literature (13 of 81 articles) examining links between dioxin or DL-PCB exposure and diabetes incidence. Furthermore, the majority of studies (45 of 81 articles) in the general population did not stratify data by sex, which limited our analysis of non-disaster exposure. Our study also revealed significant publication bias in female data, but not male data. Trim and Fill analysis implied that female-specific RR values are likely modestly overestimated in our meta-analysis. We are confident that this modest publication bias does not impact our overall conclusions about sex differences. However, the sex-specific publication bias emphasizes the need for more comprehensive representation of female data in the epidemiological literature. Additional research in sex-stratified populations is essential to confirm the sex differences revealed by our meta-analysis in disaster-exposed populations and to elucidate potential sex differences in the general population. It would also be of interest to compare circulating pollutant concentrations between sexes following disaster-exposure, as this could contribute to the sex differences found in our meta-analyses. A more comprehensive meta-analysis that considers other POPs, such as organochlorine pesticides or perfluorinated chemicals, could also facilitate a more complete picture of the literature and identify potential sex-specific associations.

Despite limitations in sex-stratified epidemiological literature, we noted parallels between the human and rodent data that strengthen our hypothesis that females are more prone to dioxin/DL pollutant-induced diabetes than males. For example, in human populations with disaster-exposure to dioxins/DL-PCBs, females had a higher risk for T2D than males. The rodent study that best models a disaster exposure scenario is a single, high-dose TCDD exposure protocol, in which TCDD-exposed female but not male mice developed transient hyperglycemia compared to vehicle-exposed controls (Hoyeck et al., 2020). To mimic the chronic low level exposure that humans experience in non-disaster settings, a repeated low-dose chemical exposure protocol in rodents is often used. Our lab reported that low-dose TCDD exposure for 12 weeks did not disrupt glucose homeostasis in male or female mice fed a chow diet (Matteo et al., 2021). However, TCDD accelerated the onset of high fat diet-induced hyperglycemia in female mice but not male mice (Matteo et al., 2021). The epidemiology data in non-disaster cohorts showed a consistent increased risk of diabetes incidence in both males and females but these studies did not consider body mass index (BMI) or diet composition, which may contribute to sex-specific associations. Another consideration is that low-dose TCDD exposure for 12 weeks in mice is far from the life-long exposure experienced by humans. So while male mice did not develop hyperglycemia in this time frame, longer-term studies are warranted given the clear epidemiological association between background level TCDD exposure and increased diabetes incidence in males.

One potential mechanism for the effects of dioxin/DL-PCB exposure on glucose homeostasis is AhR activation in relevant tissues, including pancreatic islets (Ibrahim et al., 2020). Interestingly, AhR upregulates CYP enzyme isoforms in a sex-specific manner (Kwekel et al., 2010; Yang and Li 2012) and several studies reported higher levels of CYP enzymes, including CYP1A1, CYP1A2, CYP1B1 in females compared to males in mice, pigs, and humans (Skaanild and Christian 1999; Finnström et al., 2002; Lu et al., 2013). In addition, Roh et al. (2015) reported that in humans, females had significantly higher AhR ligand activity (including both exogenous and endogenous ligands) than males. There was also a significant association between increased serum AhR ligand activity and T2D incidence (Roh et al., 2015). The AhR pathway is involved in multiple essential cellular functions, including xenobiotic metabolism, cell cycle, inflammation, circadian rhythm, adhesion and migration, cellular plasticity, and estrogen receptor signaling (Kung, Murphy, and White 2009; Swedenborg and Pongratz 2010; Anderson et al., 2013; Lu et al., 2013; Quintana and Sherr 2013; Nebert 2017; Bock 2018; Larigot et al., 2018). Exploring potential mechanisms underlying sex differences in AhR activation and CYP enzyme expression is beyond the scope of this article but deserves further study.

Age of exposure is an important confounding variable that we were unable to control for in our meta-analysis. However, most individual studies published risk estimates that had been adjusted for age. Our meta-analysis only considered adult populations, but future analysis should consider sex differences following early life exposure as well. Childhood exposure to dioxins and DL-PCBs is associated with myriad complications that can present at birth (Tawara et al., 2009; Nghiem et al., 2019), childhood (Ngwa et al., 2015; Nishijo et al., 2012; Tai et al., 2016; Tran et al., 2016; Pham et al., 2020; Wang et al., 2019; Ames et al., 2019), and subsequently impact adult health. There are also interesting sex-specific effects reported in these populations (Nishijo et al., 2012; Nguyen et al., 2018; Tai et al., 2016; Pham et al., 2020; Wang et al., 2019; Ames et al., 2019), further emphasizing the need for sex-stratified data in the epidemiology literature.

In summary, this review compared articles that examined associations between pollutant exposure from either a disaster or non-disaster setting and T2D incidence. Females showed statistically significant associations between dioxin/DL-PCB exposure and increased diabetes risk under disaster conditions, whereas males did not. More epidemiological studies with sex-stratified data are needed to confirm this observation and further investigate potential sex differences within non-disaster exposure settings. Collectively, this work will help to inform legislation and policy-makers on taking measures towards pollutant control.
